# Predictors of diarrheal mortality and patterns of caregiver health seeking behavior in in Karachi, Pakistan

**DOI:** 10.7189/jogh.6.020406

**Published:** 2016-12

**Authors:** Farah Naz Qamar, Umber Zaman, Farheen Quadri, Asia Khan, Babar Tasneem Shaikh, Iqbal Azam, Dilruba Nasrin, Karen Kotloff, Myron Levine, Nick Brown, Anita K M Zaidi

**Affiliations:** 1 Department of Paediatrics and Child Health, Aga Khan University, Karachi, Pakistan; 2 Pediatric Department, Salisbury District Hospital, Wiltshire, UK; 3 Aga Khan Foundation, Islamabad, Pakistan; 4 Department of Community Health Sciences, Aga Khan University, Karachi, Pakistan; 5 Center for Vaccine Development, Department of Pediatrics, University of Maryland School of Medicine, Baltimore, MD, USA

## Abstract

**Background:**

Pakistan is unfortunately among the five countries that contributed to the most deaths due to diarrhea and pneumonia in 2010. To explore factors associated with diarrheal deaths we assessed care–seeking behavior and other predictors of diarrhea–related mortality in children in selected low–income peri–urban communities of Karachi, Pakistan.

**Methods:**

A mixed methods study (qualitative and quantitative) using matched case–control design and focus group discussions with parents of children with moderate to severe diarrhea (MSD) was undertaken. Cases were children  <5 years of age who died within 60 days of developing an episode of MSD. Controls were age–matched children who survived after 60 days of an episode of MSD. Demographic, clinical, and care–related behavioral predictors of mortality were assessed. Conditional logistic regression was performed, matched adjusted odds ratios (mOR) are reported.

**Results:**

Parents of 77 cases and 154 controls were interviewed. Cases were less likely to receive appropriate care compared to controls (mOR = 0.2, 95% confidence interval (CI) 0.05–0.91). Refusal for hospital admission (OR = 8.9, 95% CI 2.6–30.8), and delays in reaching the health facility (OR = 3.6, 95% CI 1.0–12.9) were significant independent predictors of mortality. We found strong beliefs in traditional and spiritual healing in the population; use of both modern and traditional/spiritual treatments concurrently was common.

**Conclusion:**

Appropriate care seeking behavior predicts survival in children with diarrhea in Pakistan. There is a complex belief system relating to traditional and standard therapies. Health education for appropriate health care seeking should be implemented in order to achieve a substantial decline in diarrheal disease mortality in Pakistan.

Mortality from diarrheal diseases can be reduced dramatically, and almost eliminated if appropriate preventive and case management services are available [1]. Despite this, almost 0.578 million children under 5years die from diarrhea annually worldwide, with most deaths occurring in South Asia and sub–Saharan Africa [2,3]. A recent expert consultation identified several barriers and bottlenecks in reducing childhood diarrhea–related mortality in low–income countries [4]. These included: the absence of national coordination within ministries and other stakeholders to deliver interventions; insufficient financial resources; inadequate training and support for health workers; poor systems for monitoring and assessment of key programmatic indicators and sporadic availability of key commodities [4]. However, care–seeking behaviors by families, and their belief systems around diarrheal diseases were not identified as possible barriers, although these are well described in the literature [5].

Pakistan is a populous country of 185 million people with the fourth highest burden of child mortality globally, and insufficient progress in improving child survival [6]. Diarrhea and other infectious diseases remain major killers [7]. Low–income coastal areas of Karachi were one of seven participating sites in the, Global Enteric Multi–center Study (GEMS), a case–control study which aimed to determine the etiology, burden and adverse consequences of moderate–to–severe diarrhea (MSD), among children <5 years of age from 3 sites in Asia (Bangladesh, India, Pakistan) and 4 sites in Africa (Gambia, Kenya, Mali, Mozambique) [8,9]. One of the key findings in the GEMS was a combined odds ratio (OR) of death during the 60–day follow–up of 8.5 (95% CI 5 · 8–12 · 5) in children with MSD compared to controls without diarrhea. In Karachi the OR was the highest of all the South Asian sites at 13.1 (95% CI 0.99–172.4) [8].

To assess the high mortality at the Karachi site, we examined the potential quantitative and qualitative risk factors associated with diarrheal deaths among children under five years of age at our sites.

## METHODS

### Study population

This study was conducted at the four field sites of Karachi (Ibrahim Hyderi, Rehri Goth, Bhains Colony, and Ali Akbar Shah Goth) that participated in GEMS. The study setting has been described in detail elsewhere [9,10]. Families belong mostly to lower and middle income groups. The population at each field site had a baseline census, and maintained a demographic surveillance system (DSS). We estimated the under 5 population to be 25 094 (15.8% of total population) and the under 5 mortality to be 55 deaths per 1000 live births at the time of the study.

The data collection was done during the period of October 2009 till March 2010. Detailed methods and case definitions used in GEMS have been published elsewhere [10]. In brief, sentinel health centers were selected at each GEMS site where children from each DSS sought care when they had MSD. At the Pakistan site, we selected the Aga Khan University (AKU) Department of Pediatrics and Child Health–run primary health centers (PHC), staffed with physicians and Lady Health Workers (LHWs), that served each of the four field sites. An age stratified sample of children from the DSS who presented to the PHC with MSD during the study period were enrolled as cases. Cases underwent a standardized assessment of the demographic, epidemiologic, and clinical features at enrollment in the PHC and at a 60–day follow–up visit in the home.

### Study subjects and data collection

Two methods of investigation were conducted: a matched case-control study and a qualitative study using in depth interviews.

1. *Quantitative analysis.* For the quantitative analysis, we conducted a case-control study of children with MSD who died (cases) or survived (controls) within the DSS. Two sources of case data were used. The first source included all 16 children enrolled in GEMS who died before the 60–day follow–up visit. The second source of cases was children not enrolled in GEMS who died from an episode of diarrhea, captured by the ongoing surveillance system via a verbal autopsy conducted 30 days after death, to allow a period of mourning [11]. Two controls for each case were identified. Controls were age–matched GEMS MSD cases who survived at 60 days after the episode of MSD. Data from the standardized questionnaires performed at enrollment and follow–up during GEMS are included in the analysis, and supplemented with additional questions that were asked during interviews conducted from 2 months to 2.5 years after the death of the child. For controls the time of interview was 3 months after the episode of diarrhea. Demographic and socioeconomic variables used in GEMS were analyzed as potential covariates [12]. Appropriate care seeking behavior was defined as care sought from licensed doctors within 24 hours from the recognition of the illness (defined as the first loose stool). Health seeking behavior characteristics questioned were use of public or private transport to reach health facility, decision maker within the family, time taken to reach the health facility, number of health facilities visited and number of visits made for the episode of MSD. Correct decision to hospital admission was a composite variable created for those who either got admitted after advice or were not advised for, compared to those who refused admission after a physician’s advice.

2. *Qualitative analysis*: We conducted four focus group discussions, one at each of our study sites with the randomly selected mothers of the cases and the controls. There were total 29 participants in all the four discussions ranging from 6–9 participants in each. The duration of the sessions was from 40 minutes to one hour. Verbatim notes of focus group discussions were transcribed to provide a record of what was said. Transcription of data was done which provided a descriptive record and findings were aggregated and weighed against the quantitative results.

### Statistical methods

The sample size required used the following parameters: 80% power for detecting an estimated true effect of a magnitude of odds ratio 2.5, based on the probability of appropriate care (Po) of 30% in general population [13–15]. We estimated that 77 cases and two matched controls for each case (154 controls) were needed to fulfill the assumptions.

Conditional logistic regression was done using Statistical Analysis System (SAS) version 9.1 (SAS Institute Inc, Cary, USA). Coding was done of the descriptive data from the qualitative study and nodes and sub–nodes were formed and depending on the frequencies of responses of the participants specific themes and probes of focus group discussions were identified. Analysis was carried out by the principal investigator and the key findings were aggregated and weighed against the quantitative results.

The parent study as well as this study was approved by the Ethical Review Committee of the Aga Khan University.

## RESULTS

The majority (n = 59, 77%) of the deaths occurred in infants aged 0–11 months. The remainder were as follows: 12–23 months (n = 12, 16%) and 24–59 months (n = 7, 9%).

**Table 1** shows the social and demographic characteristics of the cases and controls. Significant differences in illness phenotype of cases and controls was observed, with 20 % of the cases having blood in stools compared to7 % of the controls (*P*< 0.005). 66% of cases had vomiting against 53% in the controls (*P*<0.12).The primary caretakers were mothers (n=215; 93%), and the relationship of the caretaker was not significantly associated with the death of a child with severe diarrhea. Formal education was universally scanty in both groups though more so in the caregivers of the fatal cases (13% vs. 27%, *P*<0.01). Every ten year increase in caretaker’s age (OR = 1.9, 95% CI 1.3–3.1) and no formal education (OR = 2.4, 95% CI 1.1–4.9) of the caretaker were independently associated with child diarrheal mortality.

**Table 1:** Baseline characteristics and the crude matched odds ratio showing associations with child diarrhea mortality

		Cases (n = 77)			Controls (n = 154)		Crude mOR	95% CI
n		%	n	%
**Characteristics of children and their illness:**								
Male		40		52	84	55		
Female		37		48	70	45	1.1	0.6–1.9
Birth order – median (IQR)		3 (2–5)			3 (2–5)		1.1	0.9–1.2
Blood in stools		15		20	11	7	3.0	1.3–7.0
Vomiting		51		66	82	53	1.7	0.9–2.9
**Caretaker’s characteristics:**								
Caretaker’s age – mean (SD)		30 (6.6)			27 (6.2)		1.9*	1.3*–3.1*
Caretaker’s education – some formal schooling		10		13	41	27		
Caretaker’s education – no formal schooling or only religious education		67		87	113	73	2.4	1.1–4.9
**Relationship of caretaker:**								
Mother		70		91	145	94		
Other than mother		7		9	9	6	1.7	0.6–4.9
**Socioeconomic and demographic characteristics:**								
Type of house material – cemented		70		91	139	90		
Type of house material – other than cement		7		9	15	10	1	0.3–2.7
Household density – median (IQR)		4 (3–6)			4 (3–6)		1.0	0.9–1.2
Number of children <5 years – median (IQR)		1 (1–2)			2 (1–2)		1.0	0.9–1.1
Number of children <5 years under care – median (IQR)		1 (1–2)			2 (1–2)		1.0	0.9–1.1
**Per capita annual income (US$):**								
Lowest		19		25	26	17		
2^nd^ quintile		19		25	31	21	0.9	0.4–2.1
Middle		13		17	28	19	0.7	0.3–1.7
4^th^ quintile		14		18	34	23	0.6	0.3–1.5
Highest		11		15	30	20	0.5	0.2–1.4
Median (IQR)		103 (62–150)			125 (82–180)			
**Water source/facility:**								
Water source – bought from tank water		15		19	44	29	1	
Water source – piped into house		48		62	102	66	2.1	0.8–5.7
Water source – public sources		14		19	8	5	9.0	2.4–34.7
Waste facility – flush toilet		13		17	51	33		
Waste facility – no flush toilet		64		83	103	67	2.7	1.3–5.5
**Asset score:**								
0–63		9		12	31	20		
64–194		25		32.5	44	29	2.2	0.8 5.7
195–336		18		23	29	19	2.3	0.9 6.2
337–958		25		32.5	50	32	1.8	0.7 4.9
Median (IQR)		195 (64–337)			195 (64–337)			
**Home care behavior of caretakers:**								
Treatment of water – boiling	13		17		83	54		
Treatment of water – not boiling	64		83		70	46	7.4	3.3–16.6
Drink offered during illness – usual or more than usual	24		31		58	37		
Drink offered during illness – less than usual	44		57		92	60	1.2	0.6–2.3
Drink offered during illness – nothing	9		12		4	3	6.1	1.5–24.5
Offered to eat during illness – usual or more than usual	14		18		34	22		
Offered to eat during illness – less than usual	50		65		110	71	1.1	0.5–2.3
Offered to eat during illness – nothing	13		17		10	7	3.7	1.2–11.5

mOR – matched adjusted odds ratios, CI – confidence interval, SD – standard deviation, IQR – interquartile range

*For every 10–year change in age.

Nearly all (92%) of the controls received appropriate care compared to 80% of the cases (**Table 2**).

**Table 2:** Healthcare seeking behavior of caretakers of children less than five years of age who died of severe diarrhea compared to those with non–fatal severe diarrhea and the crude odds ratio of the associations with diarrhea mortality

Healthcare seeking behavior characteristics	Cases (n = 77)		Controls (n = 154)		Crude mOR	95% CI
No.	%	No.	%
**Appropriate care**	62	80	141	92	0.3	0.1–0.8
**Decision maker other than mother**	10	13	7	5	3.6	1.2–10.7
**Hospital admission**:						
Correct decision	47	61	143	93		
Refused to admission	30	39	11	7	12.9	4.5–36.9
Use of public or private transport to reach health facility	22	29	40	27	1.2	0.6–2.1
**Time taken to reach health facility:**						
Less than 60 min	63	84	143	95		
60 min or more	12	16	8	5	3.2	1.3–8.3
Median (IQR)	30	15–30	25	15–30		
**Number of health facilities used:**						
1–2	67	90	145	96		
3–4	8	10	6	4	3.3	0.9–11.4
Median (IQR)	1	1–2	1	1–2		
**Number of visits:**						
1	15	20	10	7		
2 or more	60	80	141	93	0.3	0.1–0.7
Median (IQR)	3	2–4	3	2–4		

mOR – matched adjusted odds ratio, CI – confidence interval, IQR – interquartile range

On adjusted analysis (**Table 3**), children with bloody diarrhea, those whose parents refused admission to hospital or where the mother was not the decision maker were significantly more likely to die after the initial illness as were those who took longer to reach a health facility. The use of unboiled water or that from a public source additionally predicted an adverse outcome. Formal education, parental age and education did not predict outcome.

**Table 3:** Multivariable analysis of the healthcare seeking behavior for children less than five year of age who died of severe diarrhea compared to those with non–fatal severe diarrhea

	mOR	95% CI	*P–*value
**Appropriate care**	0.2	0.05–0.91	0.03
**Time taken to reach the health facility:**			
Less than one hour	1		0.04
One hour or more	3.6	1.0–12.9	
**Refusal to admission in hospital**	8.9	2.6–30.8	0.0005
**Blood in stools**	6.9	1.7–28.6	0.003
**Age of the caretaker (10 year change)**	1.6	0.8–3.4	0.21
**Education of the caretaker:**			
Some formal schooling	1		0.12
No formal schooling	2.9	0.7–11.0	
**Source of drinking water:**			
Bought	1		
Piped into the house	3.0	0.7–13.4	0.14
Public place sources	11.9	1.6–88.2	0.01
No boiling of drinking water	12.6	3.5–45.0	<0001

mOR – matched adjusted odds ratio, CI – confidence interval

### Qualitative findings

The caretaker’s knowledge and perceptions regarding diarrheal illness, knowledge regarding prevention, treatment methods, and signs of dehydration and danger signs of diarrheal death was similar in both the groups. According to 84% of the caretaker of the cases and 80% of caretaker of the controls, the treatment methods are not known. Caretaker of 84% of the cases and 78% of the control children did not know any kind of prevention methods for diarrheal illness. The majority (93%) of the caretakers of controls followed health provider advice to either admit the child on advice or not to admit if not advised compared to only 61% of caretakers of cases. Seeking spiritual treatment (called *“dum”* in the local language) was found more commonly among caretakers of fatal cases.

The results of the qualitative part of our study also highlight self–treatment at home with medicines, oral rehydration salts (ORS) and food such as yogurt and “khichri” (a traditional food which is a mixture of rice and pulses) as the main reason for the delay in seeking health care, as was deferring to family matriarchs. Although all caretakers know about giving fluids and food but they said it is difficult to offer the child usual or more than usual amount of food due to vomiting. They were also of the opinion that “*there is a treatment but it all depends on fate”*. There was almost no knowledge of prevention of AGE and little of the signs of dehydration other than that “*the eyes get smaller, the child gets weak and falls down”*. The majority agreed that ORS helps in diarrhea but some did not and a couple of caretakers of children with both fatal and non–fatal severe diarrhea were of the point of view that it helps only sometimes. One of the caretakers of a child who had died due to severe diarrhea said that *“we try our best but still we think that we cannot keep cleanliness and take good care to help our children from diarrhea”*. All the caretakers agreed that being educated can help them take better care of their children.

Additional findings from the qualitative study showed switching and simultaneous treatment from traditional, spiritual and modern healers by the caretakers of children 0–59 months with severe diarrhea was common, rather than following the advice of one provider. Limited decision making power of mothers, lack of belief in oneself for taking care of the child, inadequate knowledge of prevention of diarrhea illness, lack of awareness about water boiling, self–treating child at home resulting in delay in seeking formal care, and misconceptions regarding ORS as the factors associated more commonly with caretakers of children with severe fatal diarrhea than nonfatal diarrhea.

### DISCUSSION

The mortality from diarrheal disease in children in Pakistan remains unacceptable. All the predictors of outcome are amenable to modification. The use of unboiled water, using public water sources, travel time and blood in the stools all have potential for intervention. These findings highlight the fact that a small number of infrastructure and education interventions in the community have the potential to reduce child mortality to a great extent. Similar predictors of mortality have been reported earlier for other perinatal, neonatal and childhood illnesses [16,17].

Community education and sensitization especially a focus on women’s education will help in leveraging child health through not only better care and prevention but also through better understanding of the importance of seeking appropriate and early care for childhood illnesses. There are multiple pathways through which education of the primary care taker (who was the mother in most instances in this study), can influence child health like improved parenting, better income of the family ie, improved resources and enhanced decision making ability [17]. The other significant factor, blood in stools as a risk factor for diarrheal mortality has long been known and WHO currently recommends antibiotics for the treatment of dysentery to reduce child mortality and morbidity. Mathematical modeling data shows that >99% of the deaths due to dysentery can be averted by use of appropriate antibiotics [18]. We however have no data to comment on whether the children with dysentery who died, received antibiotic treatment or not. Other significant factors, including delayed time to reach the health facility, blood in stools, age and education of the primary caretaker, are less amenable to public health interventions.

The role of seeking care from a qualified doctor in preventing child mortality due to diarrhea illness has was also reported to be a major factor associated with infant mortality in the poorest parts of Brazil [19] in which self–treatment at home was the main reason of delay in seeking care for children. Self–treatment at home proved the main cause of delay in seeking care for children and this is compatible with results in other settings across the spectrum of common childhood illnesses [20,21]. We defined care seeking from a licensed physician as “appropriate care”, but for countries like Pakistan, with an extensive network of LHWs that are educated and entrusted to take care of relatively common illnesses such as childhood diarrhea, this criteria can be used as reference for future studies.

We found that a lack of education and women’s autonomy were major risk factors which is compatible with other studies [22–26]. If women feel insecure about their social position, they lack confidence in seeking care for their child. Moreover lack of education keeps them back from having the confidence to seek care of their children.

A strength of our study lies in its mixed method design. The qualitative research methods helped interpret the complexity of the issues [27] of health seeking behaviors which are affected by multiple factors such as social norms, culture, community dynamics, household economics, health services related factors, individuals’ experiences, context of the health facility (both geographic and social) and the inter–relation of these factors [28]. This approach allowed a rapport with mothers and families to develop, a critical element of in–depth interviewing and household visits in this particular social setting. This approach has previously been shown to enable the collection of sensitive data [29–31]. Finally, it sensitizes the community members on issues raised in the discussions (health problems, care seeking patterns and inappropriate practices) and facilitates entry of the study team into the community for quantitative surveys [32].

One potential limitation of the study is that of recall bias given that there was a delay in interviewing some parents after the child’s deaths. Recall of events for the caregivers of children with fatal severe diarrhea could be different from their comparison group in reporting child illness characteristics such as vomiting and blood in stools, health care seeking characteristics such as time to reach the health facility may have overstated facts which may bias the effect size away from the null.

However, bias is only likely to be an issue if the parents of index cases felt that hospital care would have averted the outcome which the interviews suggests was not the case. We are, therefore, confident that the direction of effect is valid.

## CONCLUSION

Appropriate care seeking behavior predicts survival in children with diarrhea in Pakistan. There is a complex belief system relating to traditional and standard therapies. Women’s autonomy and education together with availability of basic health care facilities and health education for importance of appropriate health care seeking can help to achieve a substantial decline in diarrheal disease mortality in Pakistan.

## References

[1] Chopra M, Mason E, Borrazzo J, Campbell H, Rudan I, Liu L (2013). Ending of preventable deaths from pneumonia and diarrhoea: an achievable goal.. Lancet.

[2] Liu L, Johnson HL, Cousens S, Perin J, Scott S, Lawn JE (2012). Global, regional, and national causes of child mortality: an updated systematic analysis for 2010 with time trends since 2000.. Lancet.

[3] Fischer Walker CLF, Perin J, Aryee MJ, Boschi–Pinto C, Black RE (2012). Diarrhea incidence in low–and middle–income countries in 1990 and 2010: a systematic review.. BMC Public Health.

[4] Gill CJ, Young M, Schroder K, Carvajal–Velez L, McNabb M, Aboubaker S (2013). Bottlenecks, barriers, and solutions: results from multicountry consultations focused on reduction of childhood pneumonia and diarrhoea deaths.. Lancet.

[5] Shaikh BT, Hatcher J (2005). Health seeking behaviour and health service utilization in Pakistan: challenging the policy makers.. J Public Health (Oxf)..

[6] Bhutta ZA, Hafeez A, Rizvi A, Ali N, Khan A, Ahmad F (2013). Reproductive, maternal, newborn, and child health in Pakistan: challenges and opportunities.. Lancet.

[7] National Institute of Population Studies. Islamabad Pakistan. Pakistan Demographic and Health Survey 2006–07. Available: https://dhsprogram.com/pubs/pdf/FR200/FR200.pdf. Accessed: 15 January 2016.

[8] Kotloff KL, Nataro JP, Blackwelder WC, Nasrin D, Farag TH, Panchalingam S (2013). Burden and aetiology of diarrhoeal disease in infants and young children in developing countries (the Global Enteric Multicenter Study, GEMS): a prospective, case–control study.. Lancet.

[9] Quadri F, Nasrin D, Khan A, Bokhari T, Tikmani SS, Nisar MI (2013). Health care use patterns for diarrhea in children in low–income periurban communities of Karachi, Pakistan.. Am J Trop Med Hyg.

[10] Kotloff KL, Blackwelder WC, Nasrin D, Nataro JP, Farag TH, van Eijk A (2012). The Global Enteric Multicenter Study (GEMS) of diarrheal disease in infants and young children in developing countries: epidemiologic and clinical methods of the case/control study.. Clin Infect Dis.

[11] Soleman N, Chandramohan D, Shibuya K (2006). Verbal autopsy: current practices and challenges.. Bull World Health Organ.

[12] Filmer D, Pritchett LH (2001). Estimating wealth effects without expenditure data or tears: an application to educational enrollments in states of India.. Demography.

[13] Larson CP, Saha UR, Islam R, Roy N (2006). Childhood diarrhoea management practices in Bangladesh: private sector dominance and continued inequities in care.. Int J Epidemiol.

[14] Nuruddin R, Hadden WC, Petersen MR, Lim MK (2009). Does child gender determine household decision for health care in rural Thatta, Pakistan?. J Public Health (Oxf).

[15] Sreeramareddy CT, Shankar R, Sreekumaran B, Subba S, Joshi H, Ramachandran U (2006). Care seeking behaviour for childhood illness–a questionnaire survey in western Nepal.. BMC Int Health Hum Rights.

[16] Manongi R, Mtei F, Mtove G, Nadjm B, Alegana V, Noor AM (2014). Inpatient child mortality by travel time to hospital in a rural area of Tanzania.. Trop Med Int Health.

[17] Boyle MH, Racine Y, Georgiades K, Snelling D, Hong S, Omariba W (2006). The influence of economic development level, household wealth and maternal education on child health in the developing world.. Soc Sci Med.

[18] Traa BS, Walker CLF, Munos M, Black RE (2010). Antibiotics for the treatment of dysentery in children.. Int J Epidemiol.

[19] Terra de Souza AC, Peterson KE, Andrade FMO, Gardner J, Ascherio A (2000). Circumstances of post–neonatal deaths in Ceara, Northeast Brazil: mothers' health care–seeking behaviors during their infants' fatal illness.. Soc Sci Med.

[20] McCombie SC (2002). Self–treatment for malaria: the evidence and methodological issues.. Health Policy Plan.

[21] Müller O, Traor C, Becher H, Kouyat B (2003). Malaria morbidity, treatment–seeking behaviour, and mortality in a cohort of young children in rural Burkina Faso.. Trop Med Int Health.

[22] Caldwell J, McDonald P (1982). Influence of maternal education on infant and child mortality: levels and causes. Health Policy Educ.

[23] Gupta N, Jain SK, Chawla U, Hossain S, Venkatesh S (2007). An evaluation of diarrheal diseases and acute respiratory infections control programmes in a Delhi slum.. Indian J Pediatr.

[24] Zahid GM (1996). Mother's health–seeking behaviour and childhood mortality in Pakistan.. Pak Dev Rev.

[25] Shaikh BT, Haran D, Hatcher J (2008). Women's social position and health–seeking behaviors: is the health care system accessible and responsive in Pakistan?. Health Care Women Int.

[26] Ware H (1984). Effects of maternal education, women's roles, and child care on child mortality.. Popul Dev Rev.

[27] Sofaer S (2002). Qualitative research methods.. Int J Qual Health Care.

[28] Brown SC, Stevens RA, Troiano PF, Schneider MK (2002). Exploring complex phenomena: grounded theory in student affairs research.. J Coll Student Dev.

[29] Hussain R, Lobo MA, Inam B, Khan A, Qureshi AF, Marsh D (1997). Pneumonia perceptions and management: an ethnographic study in urban squatter settlements of Karachi, Pakistan.. Soc Sci Med.

[30] Malik IA, Bukhtiari N, Good M-JD, Iqbal M, Azim S, Nawaz M (1992). Mothers' fear of child death due to acute diarrhoea: a study in urban and rural communities in Northern Punjab, Pakistan.. Soc Sci Med.

[31] Mull JD, Mull DS (1988). Mothers' concepts of childhood diarrhea in rural Pakistan: what ORT program planners should know.. Soc Sci Med.

[32] Shaikh BT, Haran D, Hatcher J, Iqbal Azam S (2008). Studying health–seeking behaviours: collecting reliable data, conducting comprehensive analysis.. J Biosoc Sci.

